# Fast Signals of Opportunity Fingerprint Database Maintenance with Autonomous Unmanned Ground Vehicle for Indoor Positioning

**DOI:** 10.3390/s18103419

**Published:** 2018-10-12

**Authors:** Yitang Peng, Xiaoji Niu, Jian Tang, Dazhi Mao, Chuang Qian

**Affiliations:** GNSS Research Center, Wuhan University, No.129, Luoyu Road, Wuhan 430079, China; yitangp@163.com or yitangp@whu.edu.cn (Y.P.); xjniu@whu.edu.cn (X.N.); moscatomao@tencent.com (D.M.); qc_gnss@whu.edu.cn (C.Q.)

**Keywords:** location fingerprint database, indoor positioning, Bluetooth fingerprints, SLAM

## Abstract

Indoor positioning technology based on Received Signal Strength Indicator (RSSI) fingerprints is a potential navigation solution, which has the advantages of simple implementation, low cost and high precision. However, as the radio frequency signals can be easily affected by the environmental change during its transmission, it is quite necessary to build location fingerprint database in advance and update it frequently, thereby guaranteeing the positioning accuracy. At present, the fingerprint database building methods mainly include point collection and line acquisition, both of which are usually labor-intensive and time consuming, especially in a large map area. This paper proposes a fast and efficient location fingerprint database construction and updating method based on a self-developed Unmanned Ground Vehicle (UGV) platform NAVIS, called Automatic Robot Line Collection. A smartphone was installed on NAVIS for collecting indoor Received Signal Strength Indicator (RSSI) fingerprints of Signals of Opportunity (SOP), such as Bluetooth and Wi-Fi. Meanwhile, indoor map was created by 2D LiDAR-based Simultaneous Localization and Mapping (SLAM) technology. The UGV automatically traverse the unknown indoor environment due to a pre-designed full-coverage path planning algorithm. Then, SOP sensors collect location fingerprints and generates grid map during the process of environment-traversing. Finally, location fingerprint database is built or updated by Kriging interpolation. Field tests were carried out to verify the effectiveness and efficiency of our proposed method. The results showed that, compared with the traditional point collection and line collection schemes, the root mean square error of the fingerprinting-based positioning results were reduced by 35.9% and 25.0% in static tests and 30.0% and 21.3% respectively in dynamic tests. Moreover, our UGV can traverse the indoor environment autonomously without human-labor on data acquisition, the efficiency of the automatic robot line collection scheme is 2.65 times and 1.72 times that of the traditional point collection and the traditional line acquisition, respectively.

## 1. Introduction

With the increasing influence of Location Based Services (LBS) in human life, such as vehicles’ navigation and social networking, the research and application of localization and navigation technology has attracted many people’s attention. As is accepted, the widespread application of Global Positioning System and Inertial Navigation System (GPS/INS) technology has solved most of the positioning problems outdoors. However, LBS users commonly spend about 70% to 90% time indoors, where Global Navigation Satellite System (GNSS) signals are blocked by buildings [1]. One of the most challenging technological problems today is that “How to get precise indoor positioning results in real time.” Among the current indoor positioning schemes, the wireless radio frequency positioning method has become a very promising and competitive technical solution by virtue of low cost and high precision. The wireless signals that can be used for location fingerprinting include Wi-Fi, Bluetooth (iBeacons), ZigBee, geomagnetism and the like [2,3,4,5].

**Figure 1:**
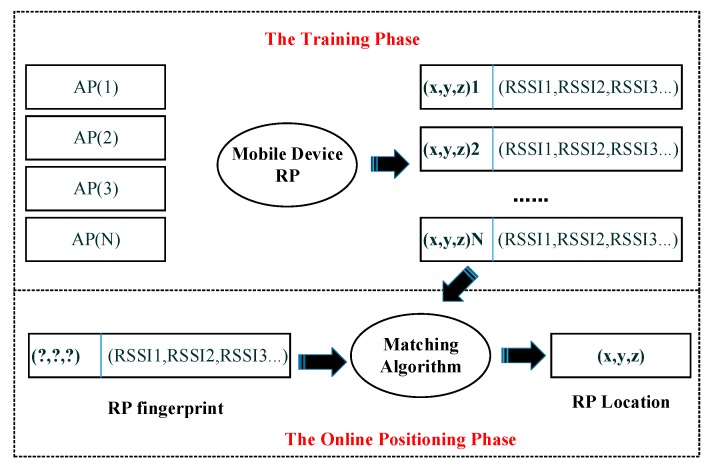
Fingerprinting-based Positioning method.

As it is shown in Figure 1, the location fingerprinting for indoor positioning system is usually achieved with two steps. Fingerprinting-based positioning is implemented in two phases: the training phase and the online positioning phase [6,7,8]. The task of the training phase is to design a sampling scheme according to the characteristics of the real indoor environment and then sampling all the sampling points in specified region with Signals of Opportunity (SOP) sensors. Finally, we can construct a location fingerprint database with recorded data, including corresponding received signal strength indicators (RSSIs), MAC address and location information, collected by sensors. In the online positioning phase, the real time Access Point (AP) signal strength and physical address information, measured by the mobile device, are utilized to match with location fingerprint database, estimating the location of the target point. Fingerprinting-based positioning technique takes advantages on low cost and high precision regardless of multipath effect and occlusion effect. However, its positioning accuracy greatly depends on the data quality of the fingerprint database so that the database need to be updated frequently due to environmental change. The establishment and maintenance of the fingerprint database seriously restricts the industrial application of the location fingerprint technology.

The traditional location fingerprinting methods mainly include point collection scheme and line collection scheme [9]. The basic idea of the traditional point collection scheme is to traverse a plurality of pre-planned reference points in a specified environment. The reference points’ positions are known in advance and each reference point approximately needs 1 min for location fingerprint collection. The traditional line collection scheme requires the mobile device to collect fingerprints back and forth along the pre-planned line segments. The coordinates of the line’s starting point and end point are known in advance and a person should hold the SOP sensors move along the line for data collection. Then, a Pedestrian Dead Reckoning (PDR) algorithm is used to match the position of the line’s end point to obtain the real-time position of the device in the moving [10]. Fingerprint database can be constructed by interpolating the real-time location and received fingerprints data. Traditional point collection and traditional line collection methods are labor-intensive, time consuming, costly and not be applicable to large areas and hazardous environments.

The difficulties on fingerprint database construction and maintenance has attracted the attention of many researchers in last decade [11,12,13]. Racko et al. [14] utilized linear interpolation or Delaunay algorithm to obtain a dense fingerprint map by interpolating the fingerprint map, which was acquired by the collected large interval points. However, the dense fingerprint map cannot be quite accurate and stable because of the data loss caused by interpolation. Tang et al. [15,16] proposed a fast fingerprint database maintenance method based on Unmanned Ground Vehicle (UGV) Simultaneous Localization and Mapping (SLAM) technology, which tests the indoor positioning accuracy by set different number of APs. On the one hand, the UGV is essentially manually controlled in reality and is still labor-intensive and time consuming. On the other hand, the paper only theoretically describes the feasibility of the fingerprint database maintenance method with UGV. Strictly speaking, this method lack reasonable quantitative analysis and database should be constructed with better interpolation method. Gu et al. [17] proposed a method by combining the Sparsity Rank Singular Value Decomposition (SRSVD) method with the K-Nearest Neighbor (KNN) algorithm to recovering absent fingerprints and reduce the workload of fingerprinting. Gunawan et al. [18] proposed a method to generate and maintain a Wi-Fi fingerprinting database automatically by using Radio Frequency Identification (RFID) tags to estimate the location of the persons, who carry the Wi-Fi scanner. Obviously, this method needs extra RFID equipment and does not efficiently reduce the workload of manual data collection.

When it is concerned about Kriging interpolation method and coverage path planning method, some previous important research results are introduced as follows: Jan et al. [19] proposed Kriging interpolation method for Wi-Fi indoor positioning system. In their paper, Kriging interpolation was used to extend the Wi-Fi database size and reduce the workload on data collection when RSSIs information are scarce. However, the errors involved in the limited RSSIs will get propagated and amplified through Kriging interpolation. The “new RSSIs data” created by Kriging interpolation will lack reliability and accuracy when the original RSSIs data are not representative or scarce. Considering these problems, the Kriging interpolation should be used to optimize existing fingerprint database instead of linearly interpolating new fingerprint data. Liu et al. [20] also proposed an improved Kriging interpolation method for fingerprint database construction. The method was mainly used to create more fingerprint data as well as Jan’s paper and also depend on human efforts for basic data collecting in essence. Ryerson et al. [21] used grid representation for farming field and a genetic algorithm was utilized to compute the optimal path traveling through all the grids. However, the research defined the grid artificially according to the environmental characteristics instead of using SLAM or other technology to generate grid map. In addition, the algorithm is designed for outdoor farming field and neglected indoor environmental features. Yang et al. [22] used neutral networks for coverage path planning. Their simulation results showed that the proposed model was capable of planning collision-free complete coverage robot paths. However, the method will be invalid in a fast changing environment, where obstacles suddenly appear in front of the robot, the neural networks will get terrible results immediately.

There are three main problems, which constrain the development and application of fingerprinting-based indoor positioning technology, summarized as follows: (1) The building and updating of fingerprint database are labor-intensive and time consuming; (2) The robot used in previous fingerprint collection methods seriously depend on manual controlling and cannot be applied to large unknown indoor environment; (3) The information matrix of SOP fingerprints database needs to be formulated and optimized with better mathematical algorithm. To address these problems, we introduce a self-designed autonomous robot platform NAVIS and utilize SLAM (simultaneous localization and mapping) algorithm to obtain accurate positioning results of the reference points and indoor grid map simultaneously. Considering the data collection method may apply to large or hazardous environment, we designed a full-coverage path planning algorithm, allowing the robot to traverse the entire unknown or known indoor environment autonomously. In addition, a smart phone was installed on the NAVIS platform to collect Bluetooth Low Energy (BLE) RSSIs in real time with our self-developed App software. Meanwhile, fingerprint database can be constructed or updated by interpolating the Bluetooth fingerprints on the position output by the SLAM algorithm. In order to test the quality of the location fingerprint database constructed by the automatic robot line collection scheme, we also did several scenario tests to compare and analyze the static and dynamic positioning accuracy of three fingerprints collection schemes and verified the scheme’s feasibility, efficiency and integrity.

The rest of this paper is organized as follow: Section 2 describes the workflow of the automatic robot line collection scheme. Section 3 designs the field tests and analyzes the experimental results and conclusions are drawn in Section 4.

## 2. Fast Fingerprinting Method Using Automatic Robot Line Collection 

### 2.1. NAVIS Hardware Platform

**Figure 2:**
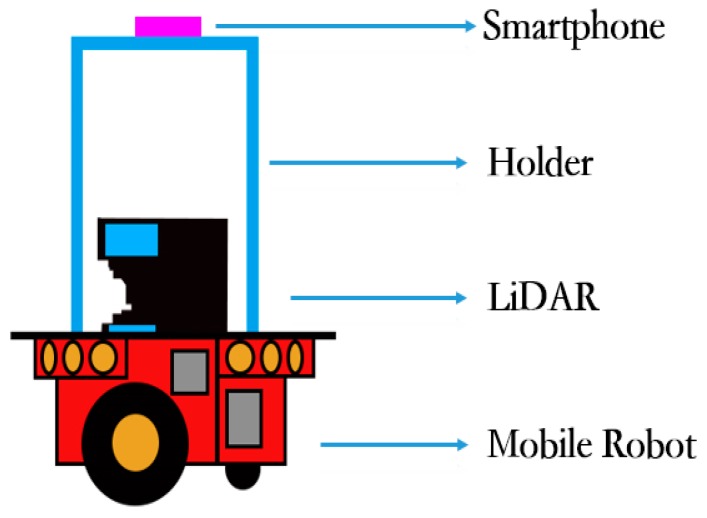
NAVIS hardware platform.

**Figure 3:**
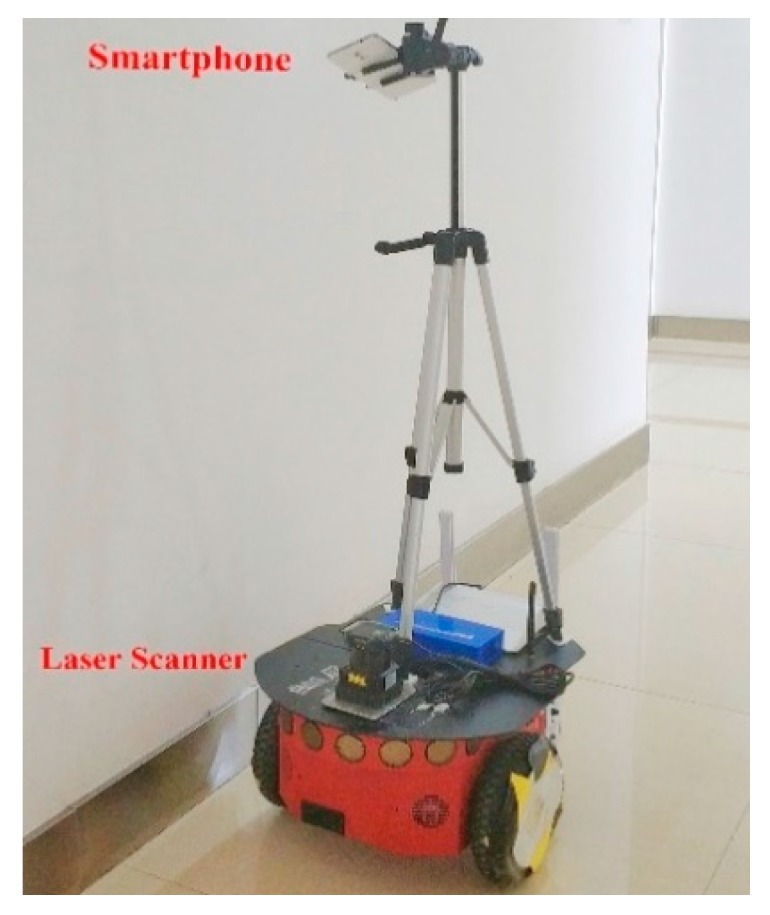
Real platform of NAVIS.

As it is shown in Figure 2 and Figure 3, the NAVIS platform mainly consists of a robot, a smartphone and a 2D laser scanner. Firstly, the robot used in this experiment is the Pioneer 3/DX pioneer robot, produced by the Mobile-Robots company, which offers Pioneer Software Development Kit (SDK) for research. The Pioneer SDK is developed based on the Robot Operating System (ROS) and can control the robot’s motion state by virtue of the serial communication. In ROS, this paper designs a full-coverage path planning algorithm, which enables the robot to explore any large unknown indoor environment autonomously. In addition, a UTM-30LX laser scanner is mounted on the robot platform for scanning indoor environment and then outputs the real-time position and grid map with the help of SLAM algorithm. Finally, a smartphone is installed on the robot platform, which is equipped with its own App software for Bluetooth RSSIs collection indoors. The App is installed on a smartphone and will collect Bluetooth RSSIs data at a frequency of 1 Hz. It is worth mentioning that the App is very simply but efficiently because of Bluetooth Module have been widely integrated into a smartphone.

### 2.2. Method Overview

**Figure 4:**
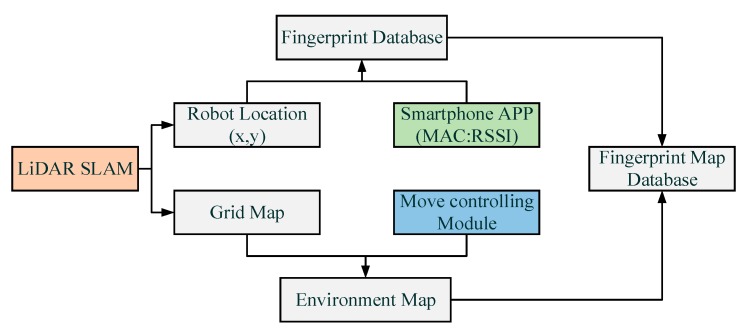
Workflow of the method.

As it is shown in Figure 4, the rapid construction method of the location fingerprint database proposed in this paper includes the following steps: (1) Designing and programming the LiDAR-based SLAM algorithm to obtain the real-time location of the robot and the current scanned grid map; (2) At the same time, the real-time location information and the fingerprint data collected by the smartphone App software will be stored in two matrix respectively and then Kriging Interpolation Algorithm is used to generate a merging matrix of fingerprint data; (3) The robot full-coverage path planning algorithm can control the robot to traverse the indoor environment autonomously, thereby generating a grid map of the entire designated area; (4) Finally, a SOP fingerprint map database can be generated by merging the fingerprint database and grid map information. The SOP fingerprint map database consists of numerous location coordinates of indoor grid map nodes and corresponding SOP measurements. 

### 2.3. Real Time LiDAR-Based SLAM Algorithm

**Figure 5:**
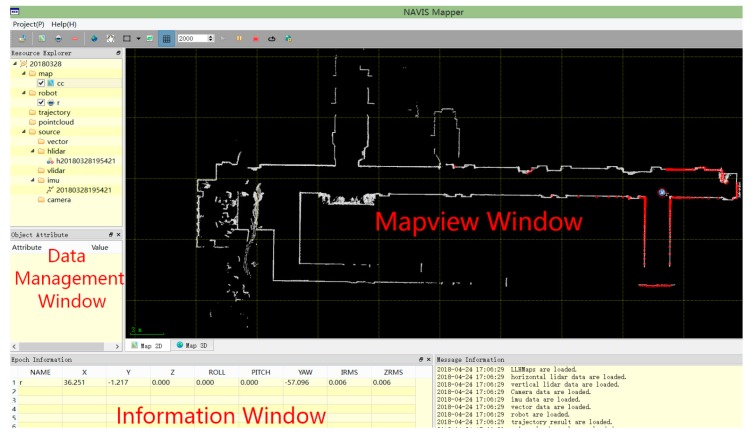
LiDAR SLAM software.

The core of the proposed fast fingerprint database construction method is to obtain the accurate position of the robot as quickly as possible during the process of data collection. As a high-efficiency and high-precision indoor positioning and mapping technology, SLAM technology provides powerful support for the robot’s positioning [23,24]. A real-time two-dimension LiDAR-based SLAM technology is utilized for the automatic robot line collection scheme. The scan-matching algorithm designed for the SLAM is called the Improved Maximum Likelihood Estimated (IMLE) based on a multi-resolution occupied grid map. The IMLE simultaneously calculates the matching results of the point-cloud-frame mode and the frame to frame mode. Excepting that, all the point cloud information contained in the proceeded map is also integrated to get optimized results. Tests showed that IMLE performs well in a given large area indoors, who can get robust and precise matching results. In this paper, a UTM-30LX laser scanner (HOKUYO Company, Osaka, Japan) is placed on the Pioneer 3/DX robot (MobileRobots Company, Burbank, CA, USA). The laser scanner has a field-of-view of 270° with 0.25° angular resolution and the maximum measurement distance is 30 m. The software output module of the LiDAR-based SLAM algorithm is shown in Figure 5. The NAVIS can achieve an average positioning accuracy of approximately RMS 10 cm within certain limits by using our SLAM algorithm.

### 2.4. Full-Coverage Traversal Algorithm for NAVIS Robot 

One of the challenging tasks in database construction is to collect the SOP fingerprints efficiently. In order to solve this problem, this paper designs a robot full-coverage path planning algorithm based on SLAM, which allows the robot to traverse all indoor area autonomously without manual controlling. The full-coverage traversal algorithm used in this paper consists of three modules: Internal Spiral Coverage (ISC) algorithm, A* algorithm and wildfire algorithm.

#### 2.4.1. ISC Algorithm

**Figure 6:**
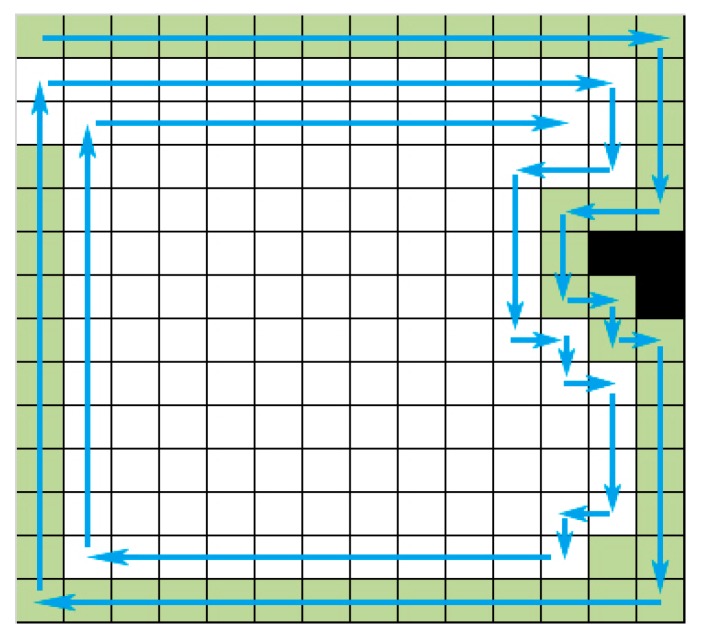
ISC traverse method.

The internal spiral coverage algorithm was proposed by Butler et al. [25]. According to Figure 6, the main idea of this method is that the mobile robot runs clockwise or counterclockwise and then traverse the whole area from initial point of the edge. For example, when the robot runs counterclockwise, if the grid on the right of the current heading direction is empty, turn right 90° and inversely if the grid on the right side of the heading direction is not empty and the forward direction grid is empty, then proceed. In the next, if there are obstacles on the right side and the forward side of current grid, then turn left 90°. According to this cycling strategy, the robot traverses the indoor environment until the right or the front side of robot’s current heading direction is not empty.

#### 2.4.2. A* Algorithm

The A* algorithm is a heuristic search algorithm based on Dijkstra algorithm, which is one of the most popular path planning algorithms. The basic idea of the A* algorithm is to judge whether the current node needs to be traversed by analyzing the actual cost from the starting point to the node and the estimated cost from the node to the end, so as to find the optimal path from the starting point to the target point. In this paper, the A* algorithm is designed for path planning of the grid map. The heuristic information *v*(*n*) is the linear distance between the current node $\left(x_{n},y_{n}\right)$ and the target node $\left(x_{W},y_{W}\right)$. Here, the Euclidean Distance is used:

(1) $$v\left(n\right)=\sqrt{{\left(x_{W}-x_{n}\right)}^{2}+{\left(y_{W}-y_{n}\right)}^{2}}$$

When the current node gets to be covered, the evaluation function *k*(*n*) can be:

(2) $$k\left(n\right)=u\left(n\right)+\sqrt{{\left(x_{W}-x_{n}\right)}^{2}+{\left(y_{W}-y_{n}\right)}^{2}}$$

where *u*(*n*) are the actual cost from starting point to the current node and *v*(*n*) are estimated cost form the current node to the end node.

The workflow of the A* algorithm for robot path planning is showed as follows:

Step1: Establish an OPEN table and a CLOSE table with the location data, add the starting point *s* to the OPEN table and add the obstacle point to the CLOSE table;
Step2: Add the node *n*, which affiliated with the smallest *k*(*n*) value in the OPEN table, in the CLOSE table;
Step3: Judge whether *n* is a target point: if *n* is a target point and then generate an optimal path according to its forward pointer; if *n* is not a target point, expanding node *n* to generate a successor node *m*;
Step4: In the OPEN table, establish a pointer from the successor node *m* to *n* and calculate *k*(*m*) = *u*(*m*) + *v*(*m*);
Step5: Add a judgment statement to determine whether there is a node *m* in the OPEN table. If the judgment fails, node *m* should be included in the OPEN table. If the judgment is successful, compare the size of the *k*(*m*) value with different forward pointers to get the smallest one;
Step6: Updating *u*(*m*), *k*(*m*) and the forward pointer of the successor node *m*;
Step7: According to the order, rearrange the *k* value in the OPEN table and return to Step 2.

In this way, the mobile robot repeatedly selects the optimal value of the evaluation function in the OPEN table and finally determines the optimal path plan. 

#### 2.4.3. The Wildfire Algorithm

**Figure 7:**
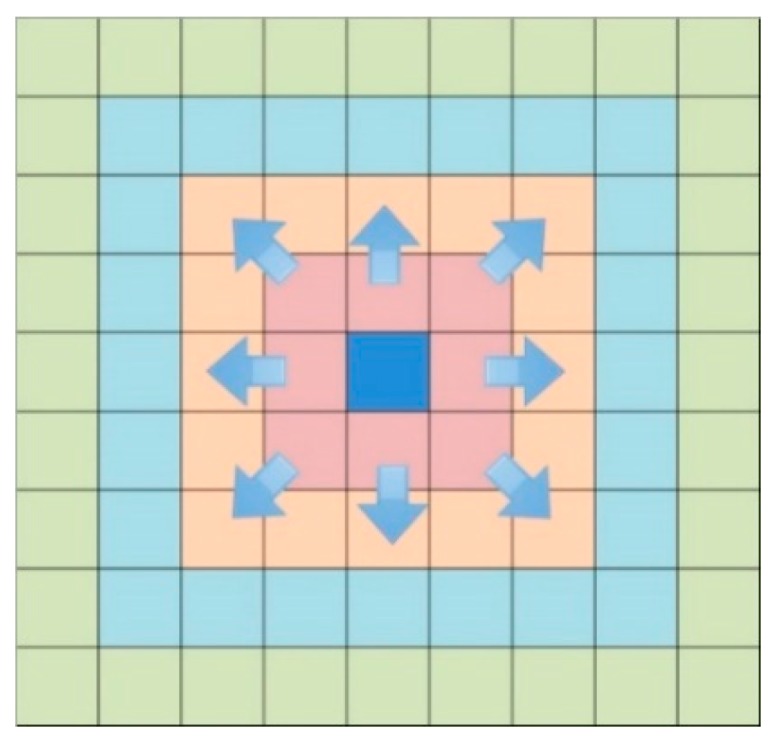
The wildfire algorithm.

The wildfire algorithm is a simple but efficient searching algorithm shown as Figure 7. The core idea of this method is to start from the starting grid and expand outwards layer by layer. Check whether each layer contains the target pre-defined grid. If not, continue to expand outward until the target grid to be found or the entire area is traversed.

**Figure 8:**
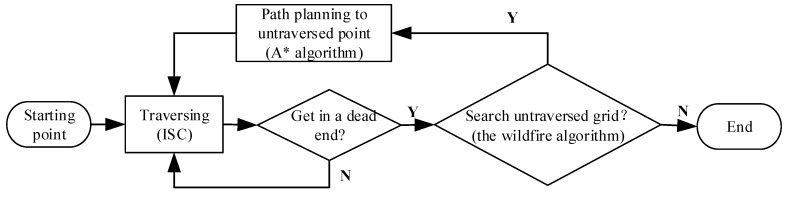
Process of the full-coverage traversal method.

In a word, based on the grid map, the full-coverage algorithm for the robot can be summarized in Figure 8. Firstly, control the robot runs from a point, which is near to the edge of grid map, at a fixed speed. Secondly, the ISC algorithm is driven to traverse the indoor environment if the robot arrives at a dead end surrounded by obstacles, the wildfire algorithm is used to search the nearest untraversed point in grid map. And then, A* algorithm can help the robot to plan an optimal path to the nearest untraversed point. The whole NAVIS system will stop until all the areas are traversed and the SOP fingerprints’ data collection is finished.

There are several advantages of the full-coverage path planning method. Firstly, the system can deal with rapidly changing environment by using ISC strategy to avoid obstacles and repeating the trajectory to get accurate fingerprints data. Secondly, the system can run autonomously and will not get into a dead end. Finally, the integrated path planning in this paper can also be used in outdoor area.

### 2.5. BLE Fingerprint Database Construction by Kriging Interpolation

Compared with the Wi-Fi signal, the Bluetooth Low-Energy (BLE) technology have wider device support and better positioning accuracy and can be set up easily. Moreover, the BLE devices iBeacons are quite cheap and portable, which enables BLE technology to be a promising solution for indoor positioning. This paper adopts iBeacons to realize indoor positioning based on location fingerprint database. The BLE location fingerprint data can be expressed by Equation as follows:

(3) $$D_{m}=\left\{(x,y,z),\left(\left({\mathrm{MAC}}_{1}:{\mathrm{RSSI}}_{1}\right),({\mathrm{MAC}}_{2}:{\mathrm{RSSI}}_{2}),\ldots ,({\mathrm{MAC}}_{n}:{\mathrm{RSSI}}_{n})\right)\right\}$$

where $D_{m}$ denotes the fingerprint data of the *m*-th point. (x,y,z) is the coordinate of $D_{m}$, ${\mathrm{MAC}}_{n}$ denotes the MAC address of the *n*-th AP, ${\mathrm{RSSI}}_{n}$ denotes received signal strength from the *n*-th AP.

As mentioned above, when the NAVIS platform traverses the specified indoor environment, the SLAM algorithm can obtain a data matrix with a one-to-one correspondence relation between time and location:

(4) $$L=\left[\begin{array}{cccc} t_{1} & x_{1} & y_{1} & z_{1}\\ t_{2} & x_{2} & y_{2} & z_{2}\\ \vdots & \vdots & \vdots & \vdots \\ t_{m} & x_{m} & y_{m} & z_{m} \end{array}\right]$$

Meanwhile, the smartphone is equipped to store BLE fingerprints information into a data matrix:

(5) $$R=\left[\begin{array}{ccccc} t_{1} & rssi_{1}^{1} & rssi_{1}^{2} & \cdots & rssi_{1}^{n}\\ t_{2} & rssi_{2}^{1} & rssi_{2}^{2} & \cdots & rssi_{2}^{n}\\ \vdots & \vdots & \vdots & \ddots & \vdots \\ t_{m} & rssi_{m}^{1} & rssi_{m}^{2} & \cdots & rssi_{m}^{n} \end{array}\right]$$

Then, the collected fingerprints data can be stored as an information matrix D:

(6) $$D=\left[\begin{array}{c} D_{1}\\ D_{2}\\ \vdots \\ D_{m} \end{array}\right]=\left[\begin{array}{cccccccc} t_{1} & x_{1} & y_{1} & z_{1} & rssi_{1}^{1} & rssi_{1}^{2} & \cdots & rssi_{1}^{n}\\ t_{2} & x_{2} & y_{2} & z_{2} & rssi_{2}^{1} & rssi_{2}^{2} & \cdots & rssi_{2}^{n}\\ \vdots & \vdots & \vdots & \vdots & \vdots & \vdots & \ddots & \vdots \\ t_{m} & x_{m} & y_{m} & z_{m} & rssi_{m}^{1} & rssi_{m}^{2} & \cdots & rssi_{m}^{n} \end{array}\right]$$

Obviously, when the robot runs in our indoor environment and collect RSSIs, the trajectory or coordinates of the robot is not exactly on the center of the grid map. A simple solution is to allocate the RSSIs collected by robot to the closest center of the grid. In this way, when robot runs fast and has big distance between two sampling point, some grids may miss relevant RSSIs. On the contrary, if the robot moves at a low speed, some grids will have superfluous RSSIs. To sum up, considerable errors will be involved in data matrix construction and cause bad positioning results in simple solution. To weaken the impact of the errors, Kriging interpolation algorithm is utilized to compute the weighted sum of several known sampling points’ RSSIs.

Matrix *D* represents the fingerprints data in a specific study area, the attribute variable is Z(p)∈D, where *p* denotes the sensor’ spatial position and $Z\left(p_{i}\right)\left(i=1,2,\ldots ,k\right)$ means the RSSIs at the sampling point $p_{i}\left(i=1,2,\ldots ,k\right)$. According to the ordinary Kriging interpolation principle, the estimated attribute value $\hat{Z}\left(p_{0}\right)$, where $p_{0}$ is an un-sampled point, is the weighted sum of the *k* known sample points’ attribute values [26]:

(7) $$\hat{Z}\left(p_{0}\right)=\overset{k}{\underset{i=1}{\sum }}\lambda_{i}Z\left(p_{i}\right)\;\lambda_{i}\left(i=1,2,\ldots ,k\right)$$

where $\lambda_{i}$ need to be calculated by other constraints.

Considering the spatial variability of wireless signal propagation, the Kriging interpolation method uses a variant function instead of a covariance function to solve the weight coefficients. When it is required to satisfy the minimum estimated variance and unbiased estimation, a system of equations for solving the weight coefficient $\lambda_{i}\left(i=1,2,\ldots ,k\right)$ can be obtained:

(8) $$\{\begin{array}{c} \sum_{i=1}^{k}\lambda_{i}\gamma \left(p_{i},p_{j}\right)-\mu =\gamma \left(p_{0},p_{i}\right)\;i=1,2,\ldots ,k\\ \sum_{i=1}^{k}\lambda_{i}=1 \end{array}$$

Define a variant function to replace a covariance function:

(9) $$\gamma \left(p_{i},p_{j}\right)=\gamma \left(p_{i}-p_{j}\right)=\frac{1}{2}E{\left[Z\left(p_{i}\right)-Z\left(p_{j}\right)\right]}^{2}$$

Then, Equation (7) satisfies the unbiased constraint as shown in Equation (10):

(10) $$E{\left[\hat{z}(p_{0})-z(p_{0})\right]}^{2}=0$$

In this paper, *k* is defined as the numbers of sampling points in a grid and the coordinate of $p_{0}$, which means the coordinate of the center of a grid, can be output by SLAM module. More important, SLAM module can get the coordinates of all center of the grids in specific. According to Kriging interpolation algorithm, we can estimate the RSSIs at the center of the grids and get a new fingerprints data information matrix:

(11) $$D^{\prime }=\left[\begin{array}{cc} p_{0}^{1} & {\mathrm{RSSIs}}^{1}\\ p_{0}^{2} & {\mathrm{RSSIs}}^{2}\\ \vdots & \vdots \\ p_{0}^{l} & {\mathrm{RSSIs}}^{l} \end{array}\right]=\left[\begin{array}{ccccccc} x_{1} & y_{1} & z_{1} & rssi_{1}^{1} & rssi_{1}^{2} & \cdots & rssi_{1}^{k}\\ x_{2} & y_{2} & z_{2} & rssi_{2}^{1} & rssi_{2}^{2} & \cdots & rssi_{2}^{k}\\ \vdots & \vdots & \vdots & \vdots & \vdots & \ddots & \vdots \\ x_{l} & y_{l} & z_{l} & rssi_{l}^{1} & rssi_{l}^{2} & \cdots & rssi_{l}^{k} \end{array}\right]$$

In Equation (11), $p_{0}^{l}(l<m)$ means the *l*-th grid’s center location and ${\mathrm{RSSIs}}_{0}^{l}(l<m)$ means estimated fingerprints data of the *l*-th grid’s center.

### 2.6. Fingerprint Matching Algorithm: WKNN

The fingerprint matching algorithm used in this paper is Weighted K-Nearest Neighbor (WKNN). The WKNN method searches for the *k* best matches of the wireless signal strength indicators to the wireless fingerprint map database and calculates the mean of the *k* weighted positions to localize the estimated position. In order to get proper weighted positions, it is critical to formulate a reliable method for weighting the influence of different positions. The location fingerprint database generally uses Euclidean Metric (EM) to evaluate the matching degree of two fingerprint feature vectors.

Define a fingerprint feature vector called ${\mathrm{RSSI}}_{i}$, which belongs to fingerprint database:

(12) $${\mathrm{RSSI}}_{i}=\left\{{\mathrm{RSSI}}_{i1},{\mathrm{RSSI}}_{i2},{\mathrm{RSSI}}_{i3},\cdots ,{\mathrm{RSSI}}_{im}\right\}$$

Define the current fingerprint feature vector named ${\mathrm{RSSI}}_{k}$, which belongs to Receive Point (RP):

(13) $${\mathrm{RSSI}}_{k}=\left\{{\mathrm{RSSI}}_{k1},{\mathrm{RSSI}}_{k2},{\mathrm{RSSI}}_{k3},\cdots ,{\mathrm{RSSI}}_{kn}\right\}$$

And then the EM distance between ${\mathrm{RSSI}}_{i}$ and ${\mathrm{RSSI}}_{k}$ can be calculated as follows:

(14) $$D\left\{{\mathrm{RSSI}}_{k},{\mathrm{RSSI}}_{i}\right\}=\sqrt{{\left({\mathrm{RSSI}}_{k1}-{\mathrm{RSSI}}_{i1}\right)}^{2}+{\left({\mathrm{RSSI}}_{k2}-{\mathrm{RSSI}}_{i2}\right)}^{2}+\cdots +{\left({\mathrm{RSSI}}_{km}-{\mathrm{RSSI}}_{im}\right)}^{2}}$$

Calculating all distances between fingerprint feature vectors ${\mathrm{RSSI}}_{i}(i=1,2,\ldots m$) in the database and the current fingerprint feature vector ${\mathrm{RSSI}}_{k}$ and then sort all $D\left\{{\mathrm{RSSI}}_{k},{\mathrm{RSSI}}_{i}\right\}$ values from small to large. 

The *k* smallest EM distance can be recorded as follows:

(15) $$D_{\min k}\left\{{\mathrm{RSSI}}_{x},{\mathrm{RSSI}}_{i}\right\}=\{D_{1}\left\{{\mathrm{RSSI}}_{x},{\mathrm{RSSI}}_{i}\right\},\cdots ,D_{k}\left\{{\mathrm{RSSI}}_{x},{\mathrm{RSSI}}_{i}\right\}\}$$

In the next, distribute different weight to these positions according to their EM distance value $D_{i}\left(i=1,2,\ldots ,k\right)$:

(16) $$p_{i}=\frac{D_{1}+D_{2}+\cdots +D_{k}}{D_{i}}$$

Finally, the current location can be calculated as following formula, where $\left(x_{i},y_{i}\right)$ means location of the *i*-th fingerprint point:

(17) $$\left(x,y\right)=\frac{1}{k}\overset{k}{\underset{i=1}{\sum }}p_{i}\left(x_{i},y_{i}\right)$$

So far, Section 2 has briefly elaborated the main algorithms or parts included in SOP fingerprint database maintenance method with autonomous UGV. After that, we will design a series of field tests in Section 3 to verify automatic robot line collection scheme.

## 3. Fields Tests and Comparative Analysis

### 3.1. Fingerprint Data Collection Schemes Overview

**Figure 9:**
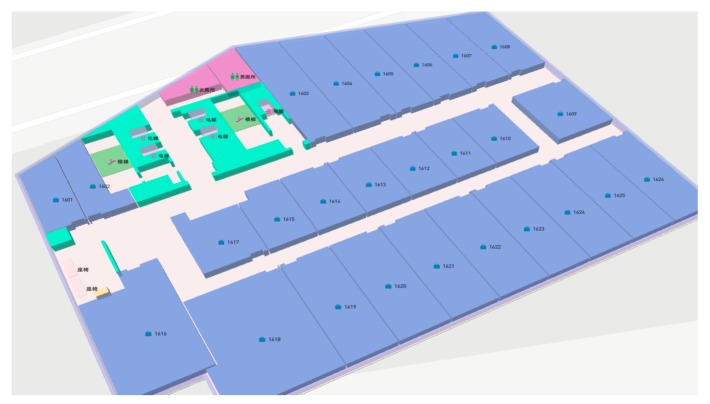
Map of the test environment.

To verify the proposed scheme, we conducted a series of indoor positioning tests on the floor with a size of $210\;m^{2}$ and Figure 9 represents the model of the environment. According to the actual scene of the floor, we designed the traditional point collection scheme, the traditional line collection scheme and the automatic robot line collection scheme.

- Traditional point collection scheme: Initially, we selected some sampling points to be collected and then manually held the smart phone to collect each static point fingerprint data at the center of selected unit. The fingerprint point acquisition time for each point is taken as 1 min.
- Traditional line collection scheme: We pre-planned several lines for data collection and each line needs to stake out in advance. Also, we measured the coordinates of the starting point and endpoint of each line selected. Finally, we used the smartphone to accept BLE location fingerprints while moving along the lines at an approximate fixed speed.
- Automatic robot line collection scheme: We used the robot to autonomously traverse the entire environment at a fixed speed (0.5 m/s), combined with the obtained SLAM grid map and the BLE location fingerprints collected by the smartphone device, the location fingerprint database can be directly interpolated and updated.

When SOP fingerprints data collection get finished, the original data will be down-sampled according to the average number of the points to ensure equal density. Then, we compared the indoor positioning accuracy of static tests and dynamic tests and operation time of three collection schemes with the same matching positioning algorithm WKNN. The positioning frequency was set 1 Hz in this paper. Finally, we analyzed the results and evaluated the feasibility and efficiency of the automatic robot line collection scheme. 

### 3.2. Static Points Positioning Tests

**Figure 10:**
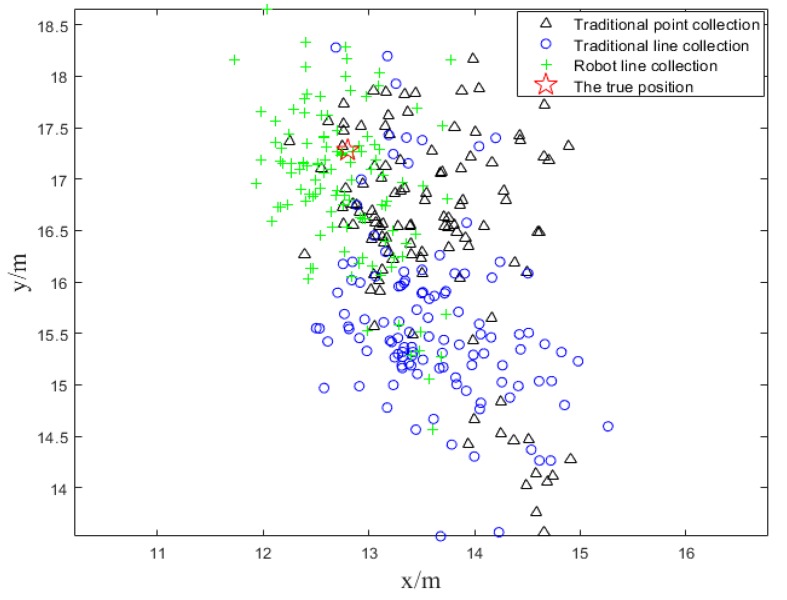
Positioning results of static test.

The main idea of static points positioning test is that we construct three BLE location fingerprint databases in advance according to above three schemes and then match the real-time SOP fingerprints data, collected by our smartphone, to our databases respectively. Firstly, we selected a typical point in the experimental environment and keep the smartphone fixed on the platform NAVIS. The reference point has the same positions in three different databases. Secondly, we put the platform on the point for about 2 min to collect enough BLE fingerprints and compare the matching positioning results. The positioning result is shown in the figures below. In Figure 10, the black, blue and green symbols represent the positioning results of the traditional point acquisition scheme, the traditional line collection scheme and the automatic robot line acquisition and the red star marks the real position.

As it is shown in Figure 10, an obvious conclusion can be drawn that the matching positioning points of automatic line collection are closer to the true position among three schemes. The black and blue symbols scattered around the true position and do not distinguish greatly from each other. 

**Figure 11:**
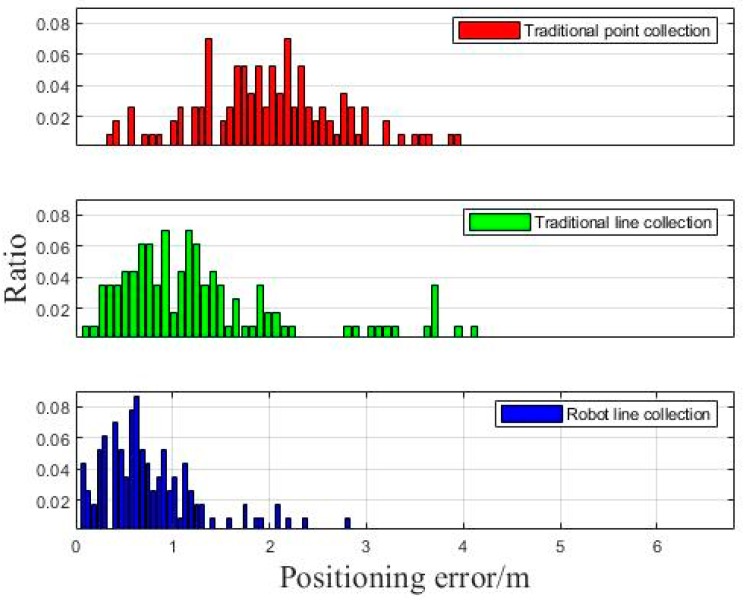
Positioning error distribution of static test.

Based on the distance between each point and the true position, we calculated the positioning error distribution of different intervals. As it is shown in Figure 11, the three colors represent the error distribution of the positioning tests of three schemes respectively. 

As it is shown in Figure 11, the ratio reflects the percentage of numbers of different positioning points’ error distance. Obviously, the blue histogram centralizes at the part of smaller values, indicating that the robot line collection method can get dense error distribution for most points and greatly restrict the accumulation of the error. 

### 3.3. Dynamic Line Positioning Tests 

In the experimental environment, we selected a typical known line segment and used the above three fingerprint databases for our dynamic tests. The basic idea of the dynamic tests is controlling the platform move along the line back and forth and then matching the real-time fingerprints collected by the smartphone to three databases. 

**Figure 12:**
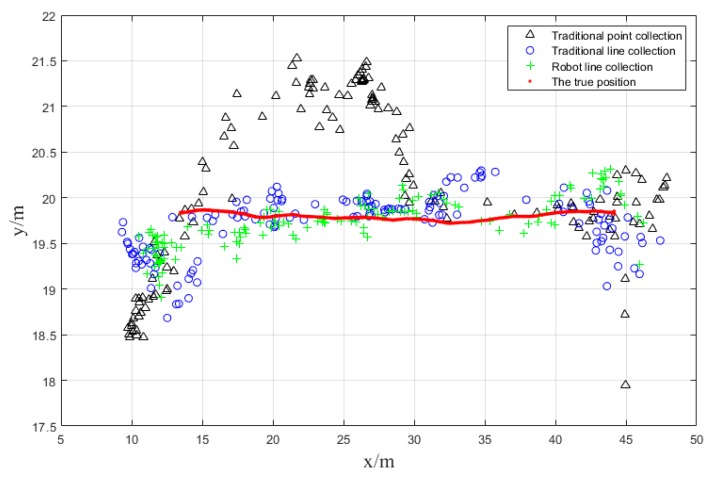
Positioning results of dynamic test.

The point map of the positioning results and the real positions are shown in Figure 12. It is quite clear that the positioning results of automatic robot line collection scheme is closest to the real trajectory of the robot, followed by the results of traditional line collection method. Unfortunately, the positioning results of traditional point collection scheme fluctuate fiercely and keep far away from the true trajectory.

**Figure 13:**
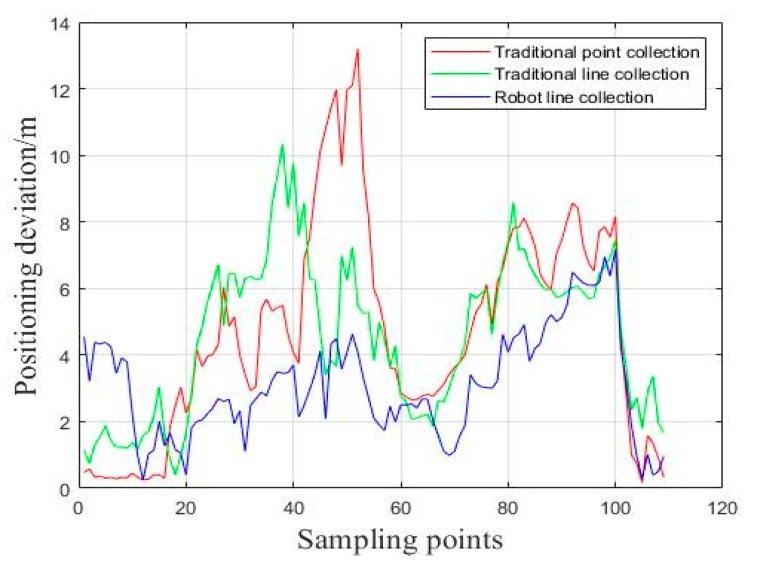
Positioning deviation of dynamic test.

The positioning results of the dynamic test can be easier understood from Figure 13, in which the three kinds of positioning results obtained by the database matching are compared with the real trajectory. The red line is the distance difference between the positioning points estimated by the traditional point acquisition method and the real positions. The green line means the positioning difference between the sampling points estimated by traditional line acquisition and the real positions and the blue line is the distance difference between the positioning coordinates by the robot line acquisition scheme and the real positions.

Comparing different lines in Figure 13, we can see that the positioning deviation between the matching positioning results of robot line collection and the true trajectory is obviously shorter than another connection lines. In other words, the real-time match-positioning results, based on the NAVIS line collection database, are closer to the real position of the robot.

**Figure 14:**
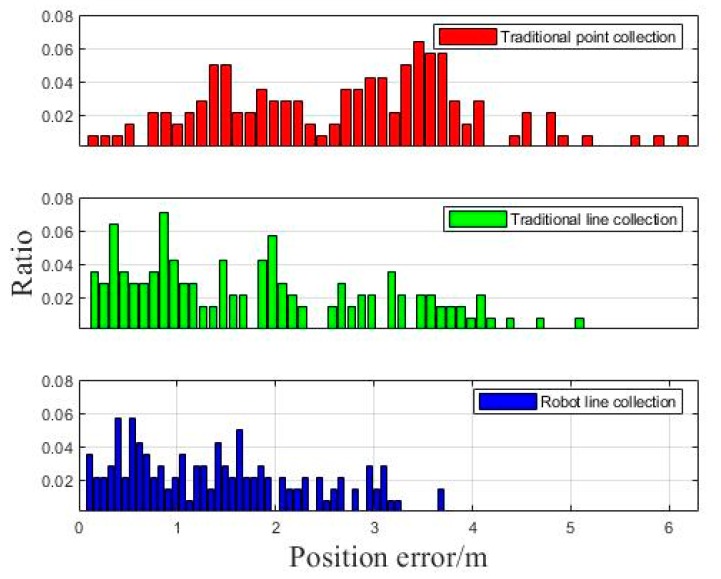
Positioning error of dynamic test.

As it is shown in Figure 14, we made a statistic analysis of the distance deviation of each point and calculated the percentage of the numbers of different positioning points’ error. 

Clearly, the robot line collection method can get dense error distribution for most points, which means that the scheme proposed in this paper can also get better positioning accuracy than traditional methods in dynamic tests.

### 3.4. Quantitative Analysis on Positioning Accuracy 

To ensure the reliability of experimental results, we selected three typical points and three typical lines for more field tests. Three static tests and three dynamic tests were conducted and the root mean square error of positioning results shown in following tables.

As it is shown in Table 1, when down-sampled, the amount of information and the fingerprint density of each fingerprint point acquired are the same, the average Root Mean Square Error (RMSE) of the static positioning results based on the automatic robot line collection method is decreased about 25.0% than that using the conventional line acquisition. Compared with the traditional point acquisition, the reduction is about 35.9%. The lowest RMSE value indicates that the location fingerprint database of the automatic robot line collection is reliable and accurate.

As it is shown in Table 2, we can clearly see that the average root mean square error (RMSE) of the dynamic positioning results in the fingerprint database obtained by the robot line acquisition method being reduced by about 21.3% compared with the conventional line acquisition, which is lower than that of the conventional point acquisition—about 30.0%. The superiority of the automatic robot line collection method to establish location fingerprint database for indoor positioning is verified.

### 3.5. Comparative Analysis of Collection Efficiency

We recorded the length of time consumed during the collection experiments. The fingerprint points collected by the three schemes have the same size and the same number (495). Traditional methods of point collection, traditional line acquisition and automatic robot line acquisition, the average number of RSSIs per fingerprint point are: 306.3, 44.6 and 79.7 and the time used to construct fingerprint database of the whole $210\;m^{2}$ area is 275 min, 26 min and 27 min respectively.

To compare the collection efficiency of three schemes, we define the acquisition efficiency as:

(18) K=α∗W/T

In the formula, *α* is the average number of RSSIs per fingerprint point; *W* is the total number of fingerprint points; *T* is the total time of the acquisition scheme, the unit is min; *K* is the collection efficiency.

**Figure 15:**
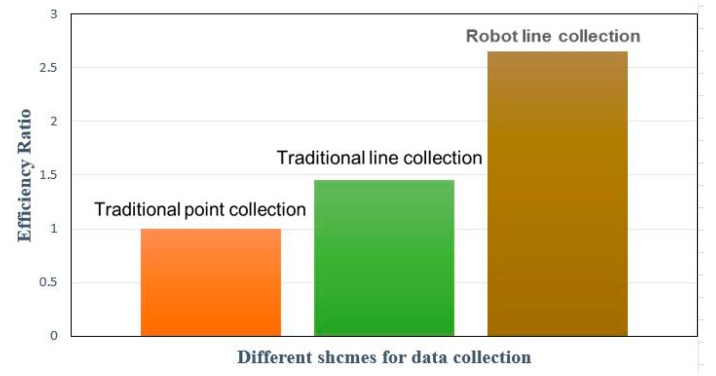
Efficiency ratio results of three schemes.

Therefore, we can get the acquisition efficiencies of the three acquisition schemes as 551.4, 849.5 and 1460.7. Based on the efficiency of the traditional point acquisition scheme, the efficiency ratio is 1:1.45:2.65 and the histogram of the collection efficiency ratio is shown in Figure 15.

The main reason for the low efficiency of the traditional point collection method and the traditional line collection scheme is that the measurement and stakeout need to be performed before collection and that the operation is discontinuous, which extends the collection work time and reduces the collection efficiency. The automatic robot line collection method can be used for continuous and uninterrupted operation, making full use of the acquisition time and having high collection efficiency. As can be seen from the above figure, the acquisition efficiency of the robot line acquisition method is 2.65 times that of the traditional point collection scheme and is 1.72 times that of the traditional line collection scheme.

In conclusion, the detailed comparison of the three schemes can be described with Table 3. According to the positioning results of a series of complex field tests, the automatic robot line collection is believed to be feasible and reliable in reality.

## 4. Conclusions and Future Works

This paper mainly proposed a fast and automatic solution for location fingerprint database maintenance. Field tests were carried out and proved the high efficiency and high reliability of the method. The main contributions of this paper are summarized as follows: (1) The autonomous UGV line collection scheme proposed in this paper is feasible, it can quickly setup and update the fingerprint database and save a lot of manpower and time cost; (2) A hybrid path planning, integrated with internal spiral algorithm, A* algorithm, the wildfire algorithm and LiDAR SLAM algorithm, was utilized successfully for full-coverage traverse of closed indoor environment; (3) The Kriging interpolation algorithm is used to construct an information matrix of location fingerprint database, which consists of the position information output by the SLAM algorithm and the signal fingerprint data collected by the smartphone; (4) The performance of the automatic UGV line acquisition method is compared with the traditional point and line acquisition methods: the RMS error of the static positioning result is reduced by 25.0% and 35.9%, the dynamic rooting result is reduced by 21.3% and 30%, respectively; (5) The efficiency of the automatic UGV line collection scheme is 2.65 times and 1.72 times that of the traditional point collection and the traditional line acquisition, respectively.

Based on the current autonomous UGV acquisition scheme, it is possible to replace the laser scanner with a camera, an efficient visual SLAM scheme is also expected for more engineering applications. Besides, the matching algorithm in this paper can also be optimized for better positioning accuracy in future work.

## Figures and Tables

**Table 1:** Positioning RMSE of three static tests.

(m)	Point Collection	Traditional Line Collection	Robot Line Collection
test 1	2.118	1.600	0.920
test 2	2.139	2.047	1.161
test 3	1.955	1.660	1.900
average	2.071	1.769	1.327

**Table 2:** Positioning RMSE of three dynamic tests.

(m)	Point Collection	Traditional Line Collection	Robot Line Collection
test 1	2.351	2.169	1.973
test 2	2.667	2.181	1.867
test 3	2.867	2.660	1.679
average	2.628	2.337	1.840

**Table 3:** Comparison of three collection schemes.

Methods	Reference Points	Time	Data Quality	Positioning Accuracy
point collection	many	longest	acceptable	worst
line collection	less	acceptable	worst	bad
robot collection	least	acceptable	best	best
